# Pathogenic Role of a Proliferation-Inducing Ligand (APRIL) in Murine IgA Nephropathy

**DOI:** 10.1371/journal.pone.0137044

**Published:** 2015-09-08

**Authors:** Yang Gyun Kim, Montserrat Alvarez, Hitoshi Suzuki, Sachiko Hirose, Shozo Izui, Yasuhiko Tomino, Bertrand Huard, Yusuke Suzuki

**Affiliations:** 1 Division of Nephrology, Department of Internal Medicine, Juntendo University School of Medicine, Tokyo, Japan; 2 Division of Nephrology, Department of Internal Medicine, Kyung Hee University School of Medicine, Seoul, Republic of Korea; 3 Department of Pathology and Immunology, University of Geneva, Geneva, Switzerland; 4 Department of Pathology, Juntendo University, School of Medicine, Tokyo, Japan; 5 Institut Albert Bonniot, INSERMU823 and Grenoble-Alpes University, La Tronche, France; Fondazione IRCCS Ospedale Maggiore Policlinico & Fondazione D’Amico per la Ricerca sulle Malattie Renali, ITALY

## Abstract

A proliferation-inducing ligand (APRIL) is a member of the tumor necrosis factor (TNF) superfamily. Despite advances in clinical and genetic studies, the details of the pathological roles of APRIL in IgA nephropathy (IgAN) remain to be fully defined. The present study aimed to further assess the pathological role of APRIL using a mouse model of IgAN. Mice with IgAN designated “grouped ddY” (gddY) were intraperitoneally administered an anti-APRIL monoclonal antibody (anti-APRIL Ab) or control IgG (Control Ab) twice each week for 2 weeks starting during the early stage of IgAN (6–7 weeks of age). Urinary albumin, serum IgA, and glomerular IgA deposition were evaluated. We further assessed the inflammatory responses during treatment by measuring the levels of the chemokine fractalkine (FKN) and its receptor CX3CR1 as well as the level of peripheral blood monocytosis. Anti-APRIL Ab treatment significantly decreased albuminuria and tissue damage combined with decreases in serum IgA levels and deposition of glomerular IgA. In contrast, the abundance of IgA^+^/B220^+^ or CD138^+^/B220^+^ B cells in the spleen and bone marrow, respectively, was unchanged. Treating gddY mice with anti-April Ab reduced the overexpression of FKN/CX3CR1 in the kidney and the increase in the population of circulating Gr1^−^/CD115^+^ monocytes. The size of the population of Gr1^−^/CD115^+^ monocytes correlated with renal FKN and urinary albumin levels. Moreover, mice treated with anti-APRIL Ab exhibited reduced progression of IgAN, serum IgA levels, and glomerular IgA deposition as well as an attenuated inflammatory process mediated by FKN-associated activation of monocytes. To the best of our knowledge, this is the first study to implicate the APRIL signal transduction pathway in the pathogenesis of nephrogenic IgA production. Moreover, our findings identify APRIL as a potential target of therapy.

## Introduction

IgA nephropathy (IgAN) is the most frequently occurring form of primary glomerulonephritis worldwide, affecting 20%–50% of patients [1]. Although IgAN was initially considered a benign glomerulonephritis, several studies have revealed its poor prognosis, variable clinical course, and pathological manifestations [2,3]. It has been reported that 30%–40% of patients with IgAN progress to end-stage renal disease within 20 years. Thus, IgAN is considered to be a major cause of end-stage renal disease in many countries [4,5].

It is widely accepted that IgA1 molecules lacking galactosyl residues in the hinge region of the heavy chain are recognized by glycan-specific antibodies and that subsequent binding forms nephritogenic IgA1–IgG immune complexes [1,6]. When these aberrantly glycosylated IgA1 and IgA1–IgG immune complexes are deposited in the glomerulus, they induce eventual renal injury. However, the underlying mechanisms of the production of nephritogenic IgA and glycan-specific IgG are not fully understood. Therefore, there are no effective treatment strategies to control the activities of nephritogenic effector molecules.

Transforming growth factor-β (TGF-β) is required for IgA production through a B cell class switch [7], and recent investigations have revealed the roles of members of the tumor necrosis factor (TNF) family, including the B-cell activation factor (BAFF) and a proliferation-inducing ligand (APRIL) [8–10]. Evidence indicates that BAFF induces a T cell-independent class switch of IgA and IgG and may therefore affect IgA production [11–13]. BAFF transgenic mice exhibit clinical phenotypes that resemble human IgAN, such as mesangial IgA deposits and high serum IgA levels in the presence of a commensal gut flora [14].

Increased IgA production by tonsillar mononuclear cells of patients with IgAN is suppressed by treatment with an anti-BAFF antibody (Ab) [15]. Moreover, recent studies have shown that the axis represented by APRIL and the transmembrane activator and calcium modulator and cyclophilin ligand interactor (TACI) play a critical role in IgA production and that the coupling between the myeloid differentiation primary response protein (MyD88) and TACI is required for B cell class switch [9,10,16,17]. Recent findings that TACI directly activates MyD88 suggest that innate immunity may be associated with the BAFF–APRIL system [18]. APRIL drives TACI-mediated switching from IgA1 to IgA2 through the Toll-like receptor (TLR) [19].

We recently established an IgAN-prone mouse model using grouped ddY (gddY) mice [20] and found that deterioration of glomerular damage in these mice is induced through mucosal activation of the TLR9/MyD88 pathway [21]. Further, we reported recently that mucosal activation of TLR9 possibly contributes to nephritogenic IgA production in patients with IgAN [21–23]. These clinical and experimental findings suggest that nephritogenic IgA production in humans and mice with IgAN is associated with the activation of APRIL–TACI during the induction of mucosal innate immunity through the TLR9/MyD88 pathway. Furthermore, dendritic cells derived from patients with lupus nephritis mediate B cell differentiation, and these B cells differentiate into IgG-secreting plasmablasts (PBs) in the presence of BAFF and IgA-secreting PBs in the presence of APRIL [24]. Moreover, serum APRIL levels in patients with IgAN are elevated significantly compared with BAFF levels [14].

Based on these findings, we hypothesized that APRIL contributes to the pathogenesis of IgAN, particularly to IgA production. However, few data are available that implicate APRIL in IgAN. A recent study found that a selective APRIL blockade in a mouse model of lupus reduces serum IgA and IgM levels and ameliorates lupus nephritis by decreasing proteinuria and renal injury [25]. Because IgAN is caused by the deposition of nephritogenic IgA in the glomerulus, we reasoned that an APRIL blockade may reduce the burden of IgA and lead to the amelioration of IgAN. To the best of our knowledge, there are no studies on the effects of inhibition of APRIL signaling on the pathogenesis of IgAN. Therefore, in this study, we used this approach to investigate the pathogenesis of IgAN in gddY mice.

## Materials and Methods

### Mice and experimental design

Type BB gddY mice [26] were raised on a diet of regular chow (Oriental Yeast, Tokyo, Japan) and water *ad libitum* in pathogen-free conditions at the animal institution of Juntendo University. Since we did not find gender difference in the anti-APRIL treatment (data not shown), we used female gddY mice in this study. The animals were randomly divided into an anti-APRIL Ab group, comprising 15 gddY mice treated with anti-APRIL monoclonal Ab [25], and a Control Ab group, comprising 10 gddY mice that received mouse IgG. We administered intraperitoneal injections of 100 μg each of anti-APRIL Ab or control IgG twice each week for 2 weeks to 6–7-week-old female type BB gddY mice. For comparisons with untreated gddY mice (n = 6), we evaluated other female control groups of mice of the same ages, Balb/c (n = 6) and HIGA (High IgA) mice (n = 6). Mating the latter with ddY mice with high serum IgA levels [27] generates progeny that serve as an IgAN-prone mouse model. Blood and urine samples were obtained before injection and 7 and 14 days later. The mice were sacrificed on day 14 after injection. The Ethics Review Committee for Animal Experimentation of Juntendo University Faculty of Medicine approved all animal experiments. Mice were euthanized using sodium pentobarbital, and appropriate efforts were made to minimize suffering.

### Evaluation of urinary albumin and serum IgA, IgG, and IgM levels

Serum IgA, IgG, and IgM levels were evaluated using sandwich ELISA kits (Bethyl Laboratories). Urine was collected for 24 h, and albuminuria was defined according to the albumin/creatinine ratio (ACR mg/g) using a DCA 2000 immunoassay system (Siemens Healthcare Diagnostics, Tokyo, Japan).

### Histological analysis

Kidney sections (3-μm thick) were fixed in 4% paraformaldehyde and stained with periodic acid–Schiff reagent, and light microscopy was used to assess histological changes. The extent of glomerular damage (glomerular pathological score) was evaluated using a previously described semiquantitative scoring system with modifications [28]. In brief, we examined 30 glomeruli per animal (n = 6 per group) and scored each aspect as follows: (i) matrix expansion: 0, absent and 1, >50% of glomeruli affected; (ii) adhesion of the capillary tuft to the Bowman’s capsule: 0, no and 1, yes; (iii) tuft numbers of proliferating mesangial cells (MCs) determined by MC >3 in 1 tuft: 0, absent; 1, 1 tuft; 2, 2 tufts; and 3, >3 tufts; (iv) capillary collapse: 0, absent; 2, segmental sclerosis; and 4, global sclerosis. The tubulointerstitial fibrosis score (TI score) was determined according to percentage fibrosis of the cortical area: 0, 0%–25%; 1, 26%–50%; and 2, >50%. The histological score was expressed as the average glomerular score in addition to the TI score.

### Immunofluorescence analysis of glomerular IgA, IgG, and F4/80 deposition

Kidneys were collected after perfusion with normal saline. Renal specimens were mounted in optimal cutting temperature (OCT) compound (Sakura Finetek, Tokyo, Japan) and stored at −80°C. Specimens embedded in OCT compound were cut into 3-μm-thick sections and fixed with acetone at −20°C for 5 min. The sections were washed with phosphate-buffered saline (PBS), blocked with a blocking agent (DS Pharma Biomedical, Osaka, Japan) for 30 min at room temperature, and then incubated with the primary Ab [phycoerythrin (PE)-conjugated goat anti-murine IgA (Santa Cruz Biotechnology, Inc), rabbit anti-murine IgG (Invitrogen, Life Technologies), and rat anti-murine F4/80 (Ab-D Serotec)] overnight at 4°C. After washing 3 times with PBS, the slides were incubated with secondary Ab for 30 min at room temperature, washed 3 times, and then mounted with a mounting medium (Dako, Tokyo, Japan). Confocal laser microscopy (Olympus Corporation, Tokyo, Japan) and a KS400 version 3.0 image analysis system (Carl Zeiss Vision GmbH, Germany) were used for semi-quantitative evaluations of glomerular IgA and IgG deposition.

### Real-time polymerase chain reaction (PCR) assays

Total RNA was extracted from kidney, spleen, and bone marrow (BM) with the TRIzol reagent (Invitrogen AG, Basel, Switzerland) and RNeasy mini kit (Qiagen). PCR was performed using Fast SYBR Green master mix (Applied Biosystems) and a 7500 real-time PCR system (Applied Biosystems). We performed quantitative, real-time PCR to determine the levels of GAPDH, fractalkine (FKN), and CX3CR1 mRNAs with the primers (Invitrogen) as follows: GAPDH: forward primer 5′-CATTGTGGAAGGGCTCATGA-3′, reverse primer 5′-TCTTCTGGGTGGCAGTGATG-3′; FKN: forward primer 5′- GGCTACCAGCACCACAAAGT-3′, reverse primer 5′-GGGTGGAGACAAGGATCTCA-3′; CX3CR1: forward primer 5′-TGAGTGACTGGCACTTCCTG-3′, reverse primer 5′-CGAGGACCACCAACAGATTT-3′. The results were quantified using a standard curve generated from analysis of serial dilutions of a reference cDNA prepared from the kidneys of type BB gddY mice, and the data were normalized to those of GAPDH mRNA.

### Flow cytometric analyses of spleen, BM, and peripheral blood cells

Mouse blood and spleen cells were collected in 5% EDTA/PBS and preincubated with anti-mouse 2.4G2 Ab for 10 min at 4°C. BM cells were collected after flushing the bones of mouse hind legs. Spleen cells were filtered through a cell strainer (22 μM; BD Falcon), and spleen RBCs were lysed with Tris–NH_4_Cl (pH 7.5) for 2 min at 37°C. Peripheral blood mononuclear cells (PBMCs) were obtained by incubating red blood cells (RBCs) with a lysing solution (BD Bioscience). Spleen and BM cells (10^6^ each) were analyzed using flow cytometry after the cells were incubated with antibodies conjugated to 3 different fluorophores, and the results were analyzed using a FACSCalibur (BD Bioscience, San Jose, CA). The Abs used to analyze spleen and BM cells were as follows: rat anti-mouse IgA conjugated to FITC (BD Bioscience), rat anti-mouse CD138 (BD Bioscience), and anti-mouse B220 (BD Bioscience). The Abs used for PBMCs were as follows: the AFS98 monoclonal anti-mouse CD115 Ab conjugated to Alexa Fluor 488 (eBioscience) and the rat RB6-8C5 monoclonal anti-mouse Ly6G Ab (anti-Gr1, BD Bioscience).

### Statistical analysis

Comparisons between groups were performed using the Mann–Whitney test. The relationships between various parameters were analyzed using Spearman rank correlation. A p-value of <0.05 was considered statistically significant. Statistical analyses were performed using SPSS software for Windows (version 20.0; SPSS, Chicago, IL). All graphs were generated using GraphPad Prism, version 5.00 for Windows (GraphPad Software)

## Results

### Depleting APRIL ameliorates murine IgAN

**Fig 1:**
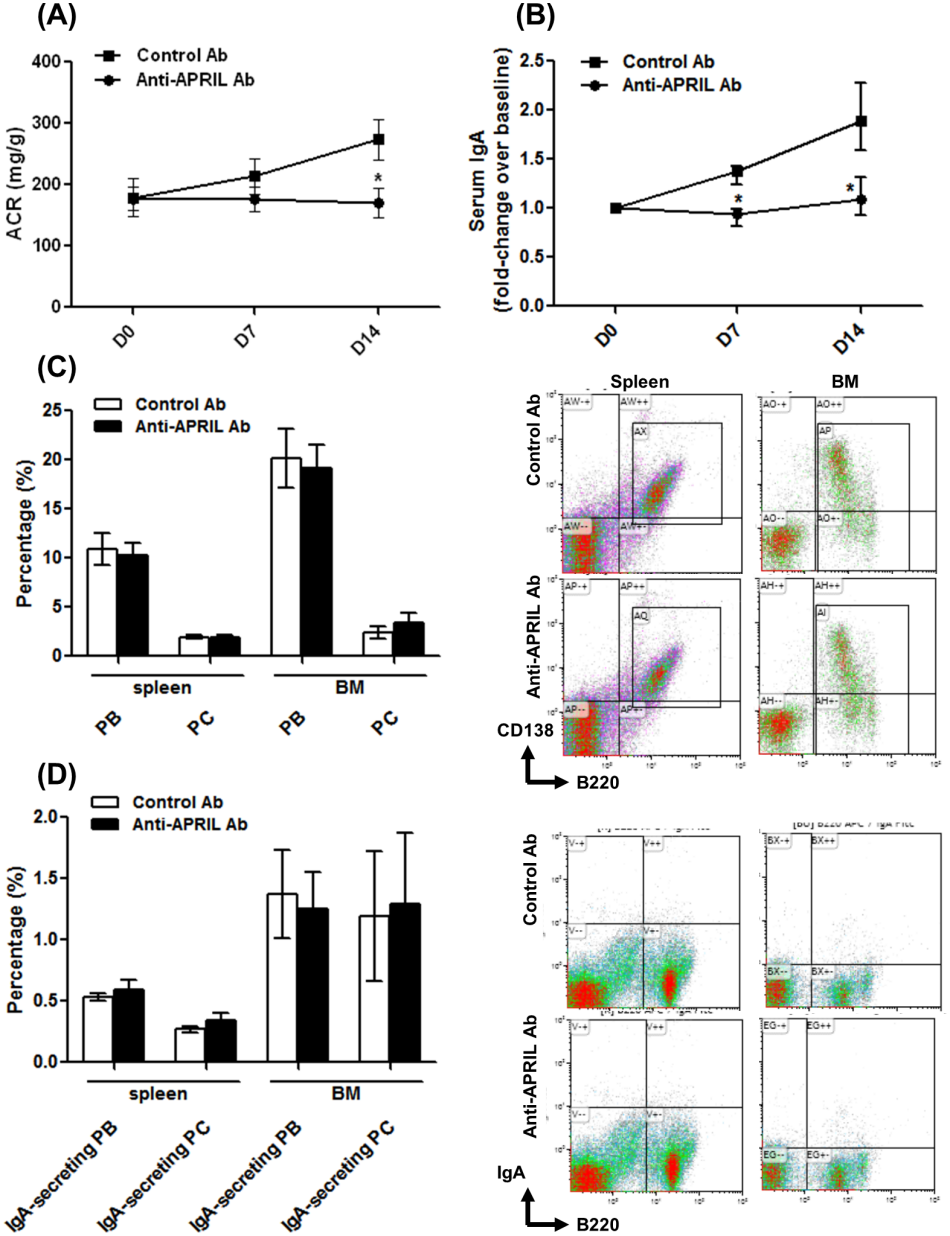
Effects of APRIL blockade on albuminuria, serum IgA levels, and B cell populations in spleen and BM. (A) The urine ACR of the anti-ARPIL Ab group differed significantly from that of the Control Ab group after 2 weeks (D14) of treatment (*p < 0.05 vs. Control Ab group). (B) The change in serum IgA levels was different from that for controls on days 7 and 14 (D7 and D14) (*p < 0.01 for D7 and p < 0.001 for D14 vs. Control Ab group). (C, D) The populations of (C) PB (CD138^+^/B220^+^) and PC (CD138^+^/B220^−^) and (D) IgA-secreting PBs (IgA^+^/B220^+^) and IgA-secreting PCs (IgA^+^/B220^−^) did not differ between the spleen and BM of each group.

We started injections of anti-APRIL Ab in the early phase of murine IgAN (6–7 weeks of age) to evaluate whether APRIL antagonism prevents disease progression. Clear clinical manifestations, including proteinuria, were observed after 8 weeks of age in type BB gddY mice [26]. Although the initial ACR values (day 0) were similar between the anti-APRIL Ab and Control Ab groups, the ACR values of the anti-APRIL Ab group differed significantly after 14 days (anti-APRIL Ab, 170.32 ± 23.91 vs. Control Ab, 273.87 ± 32.79 mg/g, p < 0.05) (Fig 1A). After 7 and 14 days, the ratios of serum IgA levels to baseline values of the anti-APRIL Ab group were significantly lower compared with those of the Control Ab group (day 7, p < 0.001; day 14, *P* < 0.001) (Fig 1B). In contrast, the ratios of serum IgG and IgM levels to baseline values were not significantly different between the groups (S1 Fig). The differences in serum IgA levels correlated positively with ACRs (*r* = 0.630, p < 0.01). Furthermore, the percentage of total PBs (CD138^+^/B220^+^) and plasma cells (PCs, CD138^+^/B220^−^) as well as IgA-secreting PBs (IgA^+^/B220^+^) and PCs (IgA^+^/B220^−^) in spleen and BM did not change significantly in either group (Fig 1C and 1D).

**Fig 2:**
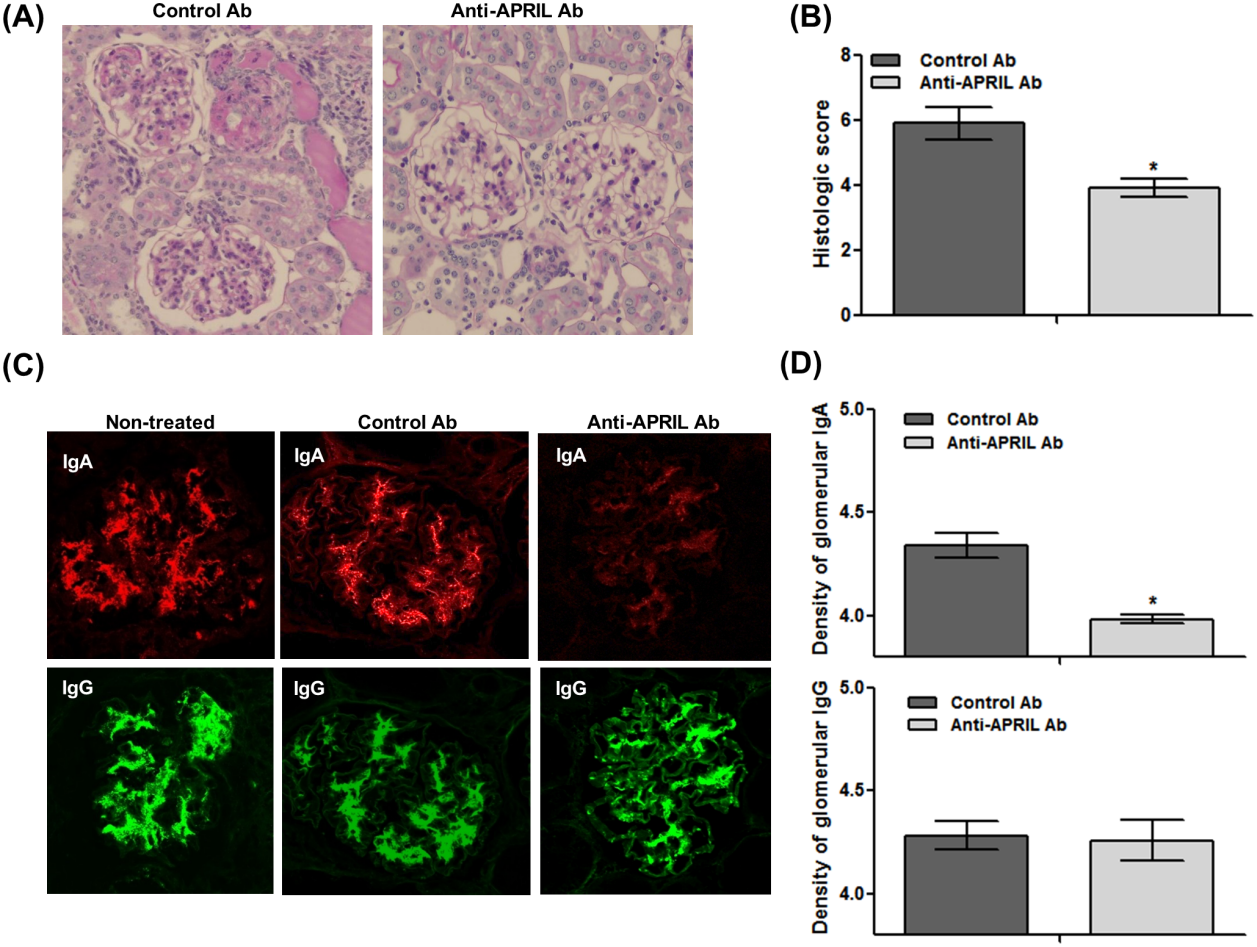
Anti-APRIL Ab ameliorates histopathological alterations and glomerular IgA deposition. (A) The pathological images of the Control Ab group (*left*) and anti-APRIL Ab group (*right*) were obtained after PAS staining (original magnification, ×200). (B) Histological analysis revealed significant attenuation of glomerular and tubulointerstitial changes in the anti-APRIL Ab group compared with the Control Ab group. (C) Representative immunofluorescence images show glomerular IgA and IgG deposition in the Control Ab group (*left*) and the anti-APRIL Ab group (*right*) (original magnification, ×400). (D) Densitometric analyses show significantly less deposition of IgA, but not IgG, in the anti-APRIL Ab group compared with the Control Ab group *p < 0.05.

Glomerular lesions, including mesangial proliferation and sclerosis with tubulointerstitial fibrosis, were attenuated in the anti-APRIL Ab group, and the histological grade was significantly lower than that of the Control Ab group (Fig 2A and 2B). Glomerular IgA and IgG depositions were evaluated using immunofluorescence analysis. Glomerular IgA depositions in the anti-APRIL Ab group were smaller than those in the Control Ab group (Fig 2C), consistent with the lack of increase of serum IgA levels in the former group. However, the staining intensity of glomerular IgG was unchanged after treatment as similar to age-matched non-treated gddY mice. Densitometric analysis confirmed that glomerular IgA deposition in the anti-APRIL Ab group was significantly lower than that in the Control Ab group (Fig 2D).

### Antagonism of APRIL function attenuates glomerular monocyte/macrophage infiltration and circulating Gr1^−^/CD115^+^ monocytes through renal FKN/CX3CR1

**Fig 3:**
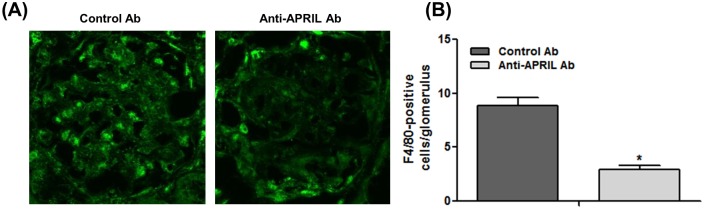
APRIL blockade reduces glomerular monocyte/macrophage infiltration. (A) Immunofluorescence analysis of F4/80 expression in the glomeruli of the Control Ab group (*left*) and the anti-APRIL Ab group (*right*) (original magnification, ×400). (B) The average number of infiltrated monocytes/macrophages per glomerulus was calculated from more than 30 glomeruli. The number of infiltrated glomerular monocytes/macrophages was decreased in the anti-APRIL Ab group. *p < 0.05.

We evaluated monocyte/macrophage infiltration as mediators of kidney injury (Fig 3A). The average number of F4/80-stained cells in glomeruli was determined using 30 glomeruli. The number of these cells decreased significantly in the anti-APRIL Ab group compared with the Control Ab group (Anti-APRIL Ab group, 8.86 ± 0.70 vs. Control Ab group, 2.93 ± 0.36, p < 0.001) (Fig 3B).

**Fig 4:**
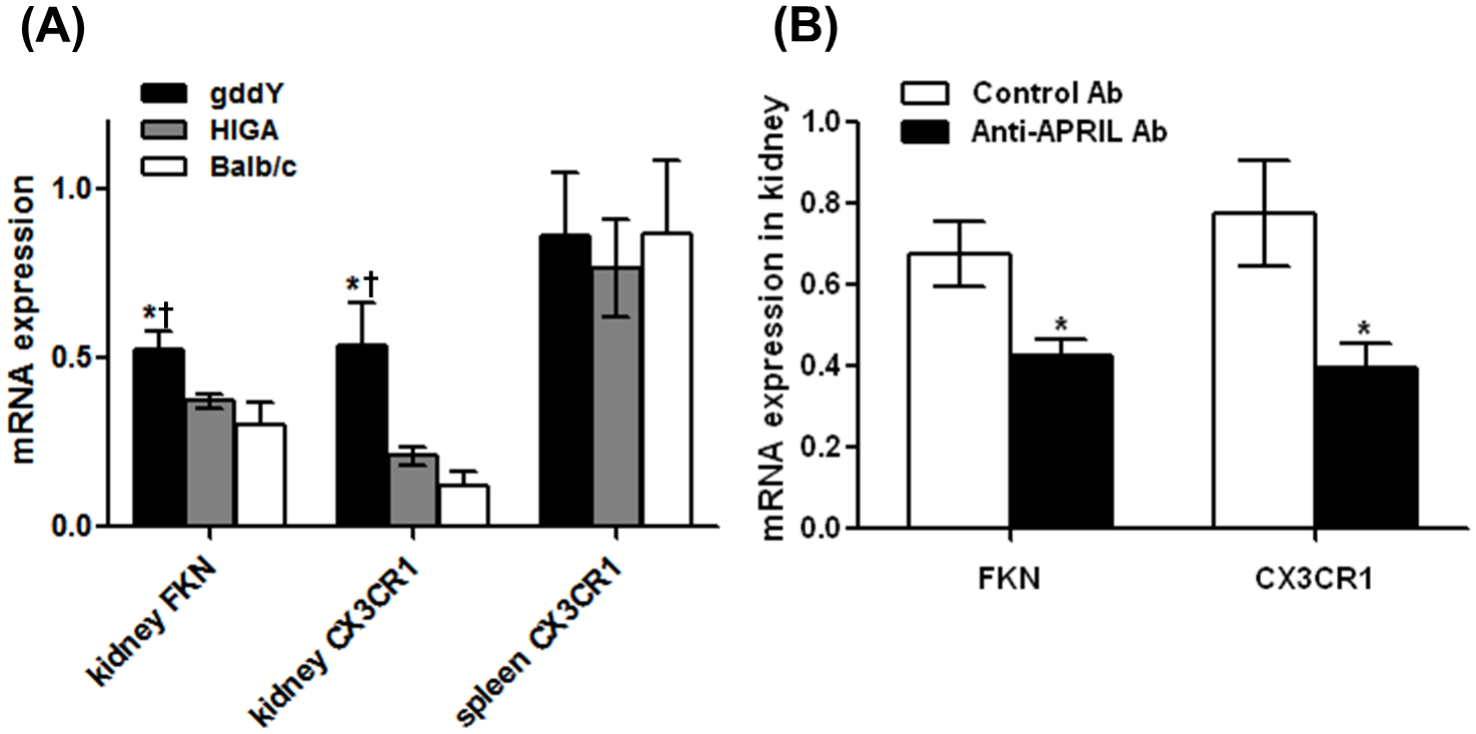
APRIL blockade is associated with attenuated renal FKN and CX3CR1 expression. (A) Real-time PCR analysis shows that renal FKN/CX3CR1 mRNA levels in 6-week-old untreated type BB gddY mice were higher than those in age-matched HIGA and Balb/c mice. (*vs. HIGA, ^†^vs. Balb/c; p < 0.01 for kidney FKN and p < 0.05 for kidney CX3CR1). In contrast, spleen CX3CR1 mRNA expression was similar in gddY, HIGA, and Balb/c mice (mRNAs levels were normalized to those of GAPDH). (B) Real-time PCR analysis of kidney samples shows that after 2 weeks of treatment, FKN and CX3CR1 mRNAs levels in the anti-APRIL Ab group were lower than those in the controls (*p < 0.05 vs. Control Ab group).

We next evaluated the levels of renal expression of the chemokine FKN and its receptor CX3CR1. After IgA deposition during IgAN, FKN triggers adhesion and transmigration of mononuclear leukocytes expressing CX3CR1 [29]. Therefore, we conducted real-time PCR analysis of FKN and CX3CR1 mRNA levels in the kidney to determine whether they were attenuated in association with the reduction of the number of mononuclear cells in the anti-APRIL Ab group. Compared with HIGA and Balb/c mice, untreated gddY mice at 6 weeks had higher renal FKN/CX3CR1 mRNA levels (ddY 0.52/0.59 vs. HIGA 0.37/0.21 vs. Balb 0.35/0.19; p < 0.05) (Fig 4A). FKN and CX3CR1 mRNA levels in the anti-APRIL Ab group after 2 weeks of treatment were significantly lower than those in the Control Ab group (p < 0.05 for both) (Fig 4B).

**Fig 5:**
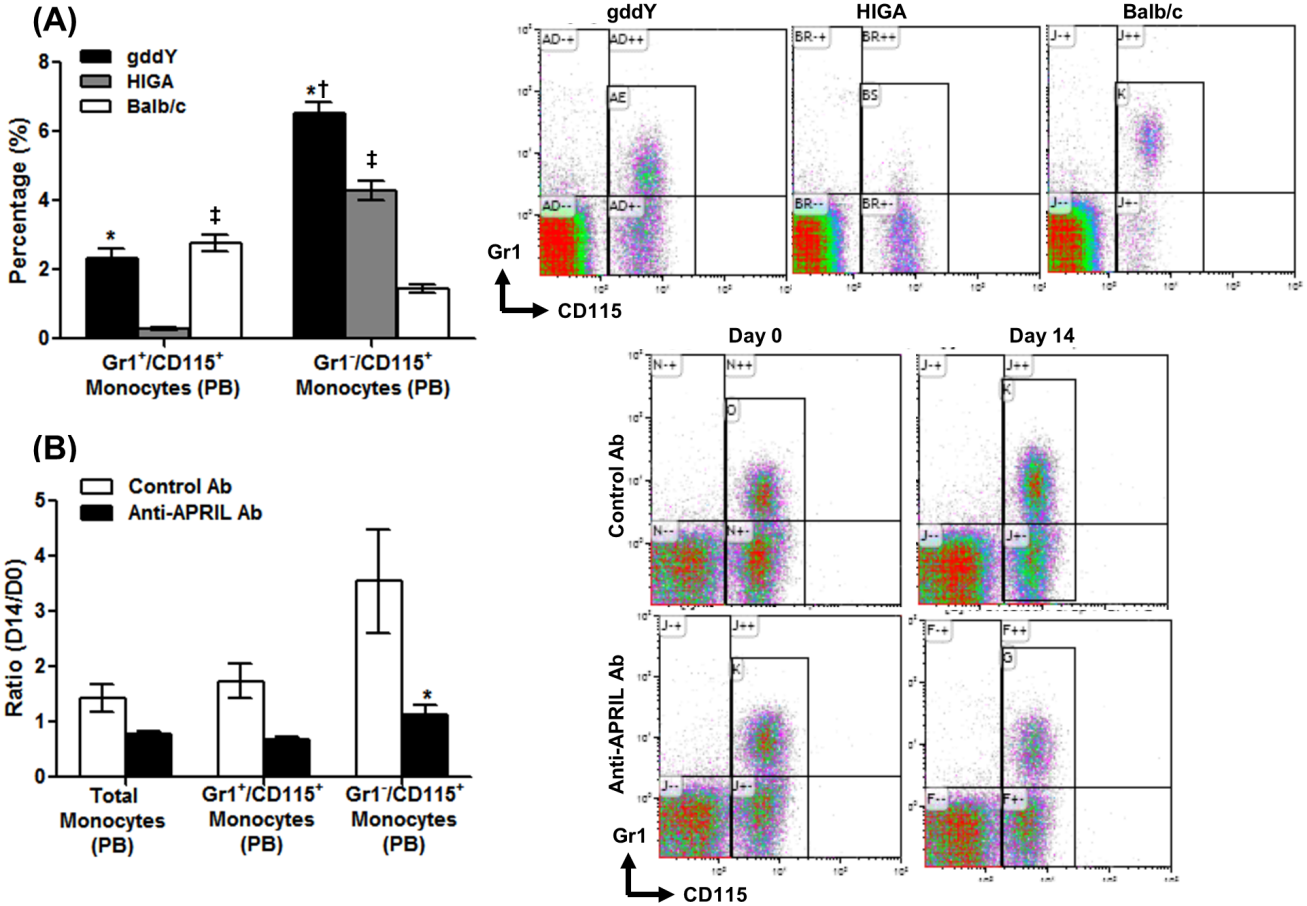
APRIL blockade affects the abundance of Gr1^−^/CD115^+^ cells among PBMCs. (A) The numbers of total monocytes, Gr1^+^/CD115^+^ monocytes, and Gr1^−^/CD115^+^ monocytes in peripheral blood were measured using flow cytometry. The percentages of Gr1^+^ and Gr1^−^ cells among PBMCs from 3 different age-matched strains of untreated mice are compared. The percentage of Gr1^−^/CD115^+^ monocytes was increased in type BB gddY mice compared with HIGA and Balb/c mice (*p < 0.001 vs. HIGA; ^†^p < 0.001 vs. Balb/c). HIGA mice had a higher percentage of Gr1^−^/CD115^+^ monocytes than Balb/c mice (^‡^p < 0.01 vs. Balb/c). The size of the population of Gr1^+^/CD115^+^ monocytes was similar between type BB gddY and Balb/c mice but was significantly lower in HIGA mice (*p < 0.001 vs. gddY, ^‡^p < 0.01 vs. Balb/c). (B) After 2 weeks (D14) of APRIL Ab treatment, the difference (D14/D0) in monocyte numbers was decreased significantly only in the Gr1^−^/CD115^+^ monocyte population (*p < 0.05 vs. Control Ab group).

We examined specific populations of monocytes, which are associated with the activation of FKN/CX3CR1 [30]. We first analyzed these monocytes in untreated mice. The number of Gr1^−^/CD115^+^ monocytes in peripheral blood (PB) was increased significantly in gddY mice compared with HIGA and Balb/c mice (gddY = 6.52%, HIGA = 4.28%, Balb/c = 1.46%, p < 0.05) (Fig 5A). Although the percentage of Gr1^+^/CD115^+^ monocytes in PB was higher in gddY mice than in HIGA mice, it was similar to that in Balb/c mice (ddY = 2.34%, HIGA = 0.29%, Balb/c = 2.77%, p > 0.05 for ddY vs. Balb/c) (Fig 5A). We next evaluated the changes in the numbers of Gr1^−^/CD115^+^ and Gr1^+^/CD115^+^ monocytes after treatment. APRIL antagonism prevented the increase in the total monocyte population from baseline (day 14 vs. day 0, p = 0.093). Moreover, the change in the number of Gr1^−^/CD115^+^ monocytes in the anti-APRIL Ab group was significantly lower than that in the Control Ab group (day 14 vs. day 0, p < 0.05). In contrast, the size of the population of Gr1^+^/CD115^+^ monocytes was unchanged after treatment (day 14 vs. day 0, p = 0.093) (Fig 5B).

**Fig 6:**
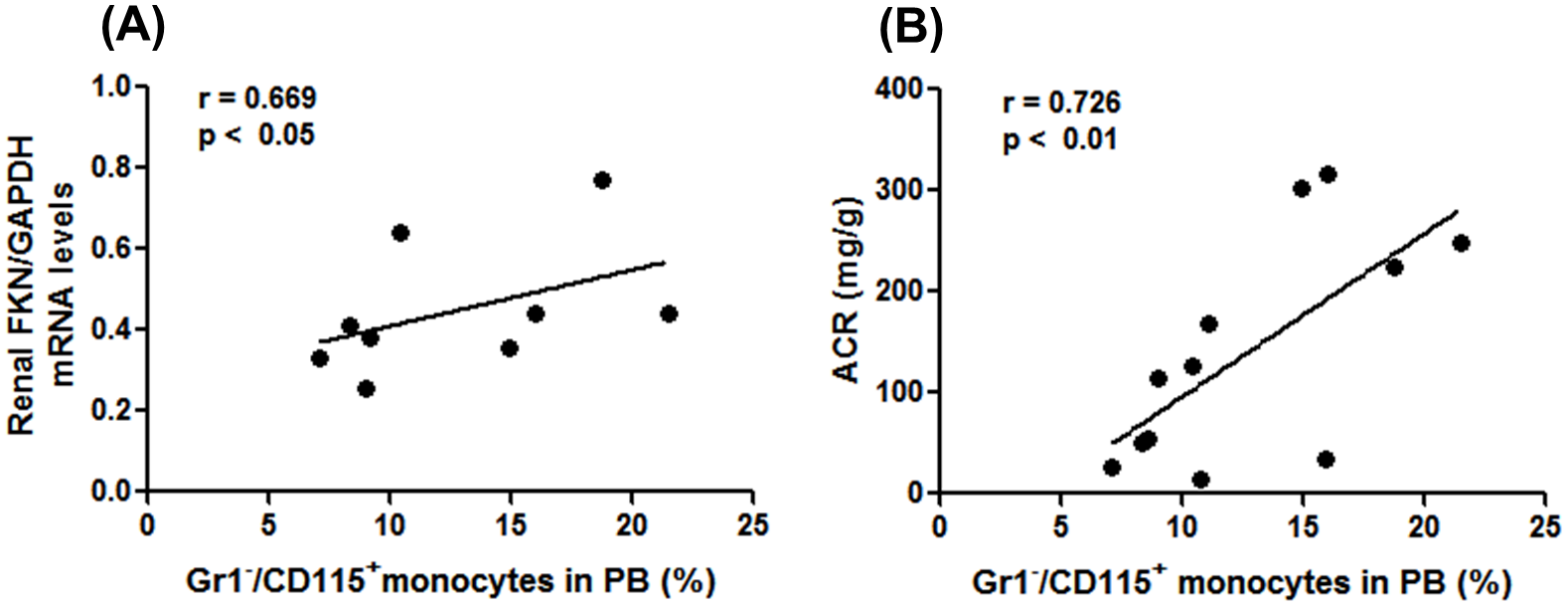
Percentage of Gr1^−^/CD115^+^ monocytes correlates with renal FKN levels and degree of albuminuria. The percentage of Gr1^−^/CD115^+^ monocytes in all mice (anti-APRIL Ab group + Control Ab group) correlated positively with (A) renal FKN mRNA expression and (B) degree of albuminuria according to Spearman rank correlation.

To further implicate the contribution of Gr1^−^/CD115^+^ monocytes to the pathogenesis of IgAN, we evaluated the correlation between the number of Gr1^−^/CD115^+^ monocytes and the inflammatory process. The percentage of Gr1^−^/CD115^+^ monocytes correlated positively with renal FKN mRNA levels (*r* = 0.669, p < 0.05) and with ACR (*r* = 0.736, p < 0.001) (Fig 6A and 6B).

## Discussion

We demonstrate here that the APRIL signaling pathway is involved in the pathogenesis of IgAN. The anti-APRIL Ab used in the present study inhibits APRIL specifically but not BAFF [25]. Moreover, establishing an APRIL blockade using the same Ab reduces proteinuria, renal damage, and serum autoantibody levels and improves the overall survival of mice with lupus [25]. We treated early-stage IgAN gddY mice with this Ab and found that it ameliorated the disease as judged by measurements of albuminuria as well as circulating and glomerular IgA levels. Selective APRIL antagonism affected only IgA levels in serum and kidney and did not induce considerable changes in the population of IgA-secreting PB or PC in BM and spleen. These findings are consistent with the results reported by a previous study, where APRIL-deficient mice had normal B cell development without a reduction in PB and PC of spleen and BM, although they showed a decrease in serum IgA levels and impaired T cell-dependent mucosal response [9]. Therefore, the present findings suggest that the APRIL blockade using the APRIL-neutralizing Ab may influence a specific B cell population producing IgA, including nephritogenic IgA, in this mouse model of IgAN.

We found previously that type BB gddY mice with IgAN have poor prognosis and that modified carbohydrate structures of IgA present in type BB gddY mice may be nephritogenic or may accelerate the formation of nephritogenic IgA-containing immune complexes [26]. Thus, increasing serum IgA levels during the course of IgAN in gddY mice in this study was expected to produce nephritogenic properties, at least in part. Moreover, we expected that the polymeric forms of serum IgA would enhance glomerular deposition via size-dependent trapping and that this trapping may be enhanced in association with pathogenic *O*-glycosylation modification of the hinge region of IgA that commonly occurs in IgAN [31]. A previous *in vitro* study has shown that interaction between mesangial IgA receptors and IgA leads to profibrotic and proinflammatory changes in mesangial cells [32–34]. Similarly, our finding that serum IgA levels correlated with ACR suggest that the increase in circulating IgA levels in the early phase of IgAN may accelerate glomerular IgA deposition and lead to subsequent renal injury. Therefore, the blockade of APRIL may reduce the production of nephritogenic IgA.

Glomerular monocyte/macrophage infiltration contributes to the pathogenesis of most types of glomerulonephritis [35]. The extent of macrophage infiltration correlates with the severity of renal injury in humans with IgAN [36]. In our present murine model of IgAN, progression of glomerular damages was associated with glomerular macrophage infiltration [28,37,38]. Moreover, we observed that attenuation of glomerular mononuclear cell infiltration in association with amelioration of proteinuria and renal lesions was induced by anti-APRIL Ab. These findings suggest pathogenic roles of glomerular monocytes/macrophages in IgAN.

Evidence acquired through studies of mouse models of lupus and diabetes indicates that FKN mediates mononuclear cell trafficking in renal disease [39,40]. Recently, the FKN/CX3CR1 axis was suggested to stimulate inflammation in humans with IgAN [29]. Gross hematuria in patients with IgAN correlates significantly with renal expression of FKN and elevation of the number of circulating CX3CR1^+^ leukocytes [29]. These findings indicate that an acute increase in glomerular IgA deposition may influence glomerular FKN expression that triggers subsequent acute inflammatory cascades, leading to the rupture of the glomerular basement membrane and subsequent leakage of proteins, red blood cells, or both.

In support of such a mechanism, we found here that FKN/CX3CR1 mRNA levels decreased significantly in the kidneys of anti-APRIL Ab-treated mice, along with a decrease in proteinuria. Moreover, renal FKN/CX3CR1 mRNA expression was significantly higher in untreated type BB gddY mice than in HIGA or Balb/c mice of the same age. HIGA mice slowly progress to IgAN, despite high serum IgA levels and large glomerular IgA deposits, suggesting a lower inflammatory capacity of glomerular IgA in HIGA mice [41]. In contrast, type BB gddY mice spontaneously develop IgAN with proteinuria and glomerular immune complex deposition by 8 weeks of age [26]. Therefore, we suggest that additional inflammatory factors, such as the oligosaccharide content of glomerular IgA or formation of immune complexes [6,26], are required for full activation of renal FKN. In the present study, in gddY mice, CX3CR1 expression was believed to be locally upregulated in the kidney, because splenic CX3CR1 expression was similar to that in other mice (HIGA and Balb/c). Therefore, the difference in FKN/CX3CR1 expression in the kidney between gddY mice and other mice was suggested to be related to the activation of renal inflammation and progression of IgAN; however, it remains unclear whether upregulated expression of renal FKN/CX3CR1 was the cause or result of the disease.

Increased FKN expression in the glomerulus and expression of CX3CR1 by glomerular CD16^+^ monocytes in murine proliferative lupus nephritis indicates that FKN induces homing of CX3CR1-positive mononuclear cells [40]. Circulating monocytes in mice comprise 2 phenotypically different subsets. The “inflammatory” monocytes express Gr1 (Gr1^+^) [32], and the “resting” or “patrolling” monocytes express CX3CR1 but not Gr1 (Gr1^−^) [42,43]. The patrolling monocytes rapidly invade infected and damaged tissues, including tissues with Ab/immune complex deposits [30]. Thereafter, these patrolling monocytes initiate the innate immune response, differentiate into macrophages, and acutely phagocytose immune complexes [30].

We show here that only the number of Gr1^−^ monocytes was significantly elevated in gddY mice compared with HIGA and Balb/c mice, supporting the idea that disease progression in gddY mice may involve activation of the renal FKN/CX3CR1 axis and the recruitment of Gr1^−^ monocytes. We observed that the APRIL blockade significantly reduced the population of CD115^+^ monocytes, particularly Gr1^−^/CD115^+^ monocytes. Furthermore, the number of circulatory patrolling monocytes (Gr1^−^/CD115^+^) correlated positively with renal FKN expression and the degree of albuminuria. These findings indicate that APRIL antagonism reduced nephritogenic IgA production, which reduced glomerular IgA deposition and led to the downregulation of FKN/CX3CR1 expression. Therefore, reduced levels of glomerular IgA and lack of peripheral increases in Gr1^−^ patrolling monocytes in gddY mice with an APRIL blockade, in part, led to amelioration of renal inflammation and subsequent proteinuria.

In conclusion, to the best of our knowledge, the present study is the first to show that APRIL antagonism ameliorated IgAN in a mouse model. Thus, serum and kidney IgA levels, including nephritogenic IgA and proteinuria, were reduced, along with reductions in renal FKN/CX3CR1 expression and circulating Gr1^−^ monocyte levels. The present data suggest that APRIL-dependent IgA production contributes to the pathogenesis of IgAN, and thus, APRIL antagonism may represent a new therapeutic approach for treating IgAN.

## Supporting Information

S1 Fig Selective APRIL blocking effects on serum IgG and IgM. The change in serum IgG and IgM was not different between anti-APRIL Ab group and controls (p > 0.05).
(DOCX)
