# Supplementary material for: Pathogenic Role of a Proliferation-Inducing Ligand (APRIL) in Murine IgA Nephropathy
Source: PLoS One. 2015 Sep 8;10(9):e0137044. doi: 10.1371/journal.pone.0137044 (PMC4562625; doi:10.1371/journal.pone.0137044)
Supplement: S1 Fig — The change in serum IgG and IgM was not different between anti-APRIL Ab group and controls (p > 0.05). (DOCX) [file pone.0137044.s001.docx]

**S1 Fig. Selective APRIL blocking effects on serum IgG and IgM**

The change in serum IgG and IgM was not different between anti-APRIL Ab group and controls (p > 0.05).

**
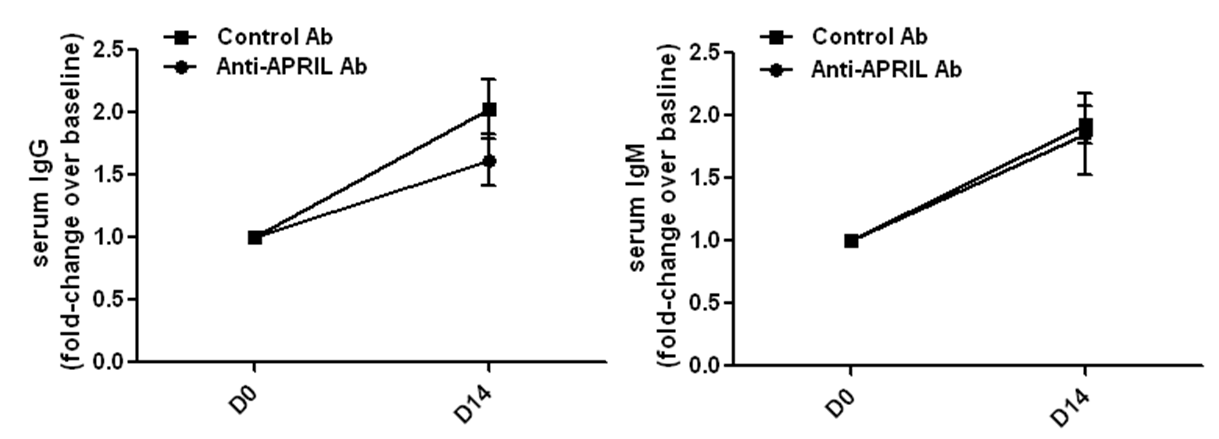
**
